# Using Prior Information from the Medical Literature in GWAS of Oral Cancer Identifies Novel Susceptibility Variant on Chromosome 4 - the AdAPT Method

**DOI:** 10.1371/journal.pone.0036888

**Published:** 2012-05-25

**Authors:** Mattias Johansson, Angus Roberts, Dan Chen, Yaoyong Li, Manon Delahaye-Sourdeix, Niraj Aswani, Mark A. Greenwood, Simone Benhamou, Pagona Lagiou, Ivana Holcátová, Lorenzo Richiardi, Kristina Kjaerheim, Antonio Agudo, Xavier Castellsagué, Tatiana V. Macfarlane, Luigi Barzan, Cristina Canova, Nalin S. Thakker, David I. Conway, Ariana Znaor, Claire M. Healy, Wolfgang Ahrens, David Zaridze, Neonilia Szeszenia-Dabrowska, Jolanta Lissowska, Eleonóra Fabiánová, Ioan Nicolae Mates, Vladimir Bencko, Lenka Foretova, Vladimir Janout, Maria Paula Curado, Sergio Koifman, Ana Menezes, Victor Wünsch-Filho, Jose Eluf-Neto, Paolo Boffetta, Silvia Franceschi, Rolando Herrero, Leticia Fernandez Garrote, Renato Talamini, Stefania Boccia, Pilar Galan, Lars Vatten, Peter Thomson, Diana Zelenika, Mark Lathrop, Graham Byrnes, Hamish Cunningham, Paul Brennan, Jon Wakefield, James D. Mckay

**Affiliations:** 1 Section of Genetics, International Agency for Research on Cancer (IARC), Lyon, France; 2 GATE team, Department of Computer Science, The University of Sheffield, Sheffield, United Kingdom; 3 Paterson Institute for Cancer Research, University of Manchester, Manchester, United Kingdom; 4 INSERM U946, Paris, France; 5 CNRS UMR8200, Gustave Roussy Institute, Villejuif, France; 6 Department of Hygiene, Epidemiology and Medical Statistics, University of Athens School of Medicine, Athens, Greece; 7 Institute of Hygiene and Epidemiology,1st Faculty of Medicine, Charles University in Prague, Prague, Czech Republic; 8 University of Turin, Unit of Cancer Epidemiology, Turin, Italy; 9 Cancer Registry of Norway, Oslo, Norway; 10 Institut Català d'Oncologia (ICO), IDIBELL, L'Hospitalet de Llobregat, Catalonia, Spain; 11 CIBER Epidemiología y Salud Pública (CIBERESP), Madrid, Spain; 12 School of Medicine and Dentistry, University of Aberdeen, Aberdeen, United Kingdom; 13 General Hospital of Pordenone, Pordenone, Italy; 14 Department of Molecular Medicine, University of Padova, Padova, Italy; 15 MRC-HPA Centre for Environment and Health, Respiratory Epidemiology and Public Health, National Heart and Lung Institute, Imperial College, London, United Kingdom; 16 School of Dentistry, University of Manchester, Manchester, United Kingdom; 17 University of Glasgow Dental School, Glasgow, Scotland, United Kingdom; 18 Croatian National Cancer Registry, Croatian National Institute of Public Health, Zagreb, Croatia; 19 Trinity College School of Dental Science, Dublin, Ireland; 20 Institute for Epidemiology and Prevention Research (BIPS), Bremen, Germany; 21 Institute for Statistics, Bremen University, Bremen, Germany; 22 Institute of Carcinogenesis, Cancer Research Centre, Moscow, Russian Federation; 23 Department of Epidemiology, Institute of Occupational Medicine, Lodz, Poland; 24 Department of Cancer Epidemiology and Prevention, M. Sklodowska-Curie Memorial Cancer Center and Institute of Oncology, Warsaw, Poland; 25 Regional Authority of Public Health, Banská Bystrica, Slovak Republic; 26 University of Medicine and Pharmacy Carol Davila, Bucharest, Romania; 27 Department of Cancer Epidemiology and Genetics, Masaryk Memorial Cancer Institute, Brno, Czech Republic; 28 Palacky University, Olomouc, Czech Republic; 29 International Prevention Research Institute (IPRI), Ecully, France; 30 Hospital Araujo Jorge da ACCG, Goias, Brazil; 31 National School of Public Health/FIOCRUZ, Rio de Janeiro, Brazil; 32 Universidade Federal de Pelotas, Pelotas, Brazil; 33 Universidade de Sao Paulo, Sao Paulo, Brazil; 34 The Tisch Cancer Institute Mount Sinai School of Medicine, New York, New York, United States of America; 35 Section of Infections, International Agency for Research on Cancer (IARC), Lyon, France; 36 Instituto de Investigación Epidemiológica, San José, Costa Rica; 37 Institute of Oncology and Radiobiology, Havana, Cuba; 38 National Cancer Institute, IRCSS, Aviano, Italy; 39 Institute of Hygiene, Università Cattolica del Sacro Cuore, Rome, Italy; 40 IRCCS San Raffaele Pisana, Rome, Italy; 41 INSERM U557 (UMR Inserm; INRA; CNAM, Université Paris 13), Paris, France; 42 CRNH IdF, Bobigny, France; 43 Norwegian University of Science and Technology, Trondheim, Norway; 44 Dental School, Newcastle University, Newcastle, United Kingdom; 45 Centre National de Génotypage, Institut Génomique, Commissariat à l'énergie Atomique, Evry, France; 46 Fondation Jean Dausset-CEPH, Paris, France; 47 Department of Biostatistics and Department of Statistics, University of Washington, Seattle, Washington, United States of America; The University of Texas M. D. Anderson Cancer Center, United States of America

## Abstract

**Background:**

Genome-wide association studies (GWAS) require large sample sizes to obtain adequate statistical power, but it may be possible to increase the power by incorporating complementary data. In this study we investigated the feasibility of automatically retrieving information from the medical literature and leveraging this information in GWAS.

**Methods:**

We developed a method that searches through PubMed abstracts for pre-assigned keywords and key concepts, and uses this information to assign prior probabilities of association for each single nucleotide polymorphism (SNP) with the phenotype of interest - the Adjusting Association Priors with Text (AdAPT) method. Association results from a GWAS can subsequently be ranked in the context of these priors using the Bayes False Discovery Probability (BFDP) framework. We initially tested AdAPT by comparing rankings of known susceptibility alleles in a previous lung cancer GWAS, and subsequently applied it in a two-phase GWAS of oral cancer.

**Results:**

Known lung cancer susceptibility SNPs were consistently ranked higher by AdAPT BFDPs than by p-values. In the oral cancer GWAS, we sought to replicate the top five SNPs as ranked by AdAPT BFDPs, of which rs991316, located in the *ADH* gene region of 4q23, displayed a statistically significant association with oral cancer risk in the replication phase (*per-rare-allele log additive p-value [p_trend_]* = 2.5×10^−3^). The combined OR for having one additional rare allele was 0.83 (95% CI: 0.76–0.90), and this association was independent of previously identified susceptibility SNPs that are associated with overall UADT cancer in this gene region. We also investigated if rs991316 was associated with other cancers of the upper aerodigestive tract (UADT), but no additional association signal was found.

**Conclusion:**

This study highlights the potential utility of systematically incorporating prior knowledge from the medical literature in genome-wide analyses using the AdAPT methodology. AdAPT is available online (url: http://services.gate.ac.uk/lld/gwas/service/config).

## Introduction

Risk effects of common susceptibility variants of complex disorders - including most cancers - are generally small (i.e. OR<1.5) [1] and genome-wide association studies (GWAS) require a stringent significance threshold (e.g. p-value<10^−7^) due to the burden of multiple testing. Thus, GWAS for cancer risk require large sample sizes in order to have sufficient statistical power. It is therefore problematic to conduct GWA studies of less common cancers for which recruiting an adequate number of cases is difficult. There may be benefit in incorporating additional evidence gathered through complementary experiments or other sources of information. Such information can be incorporated with GWAS results using simple Bayesian methods [2] for instance the method developed by Wakefield [3]. This uses the approximate Bayes factor (ABF), estimated using beta estimates and standard errors of gene variant to disease associations, together with the prior odds for the null hypothesis to generate the Bayes False Discovery Probability (BFDP). Hence, the BFDP provides an estimate of the probability that the observed result represents a false positive association, and can be used in place of p-values when ranking or otherwise evaluating association results. The main difficulty in implementing such an approach in GWAS is assigning relevant and realistic prior probabilities of association with disease for each investigated single nucleotide polymorphism (SNP).

Potential prior information for gene-disease relationships can be retrieved from various sources, for example expression quantitative trait loci (eQTL) experiments, pathway ontology databases, and literature scans [2]. In recognizing that a large number of susceptibility variants identified through GWAS reside near plausible candidate genes [4], we hypothesized that it is possible to extract prior knowledge from the text-based medical literature in order to increase the statistical power of detecting susceptibility SNPs for which such information is available.

In order to evaluate the feasibility and potential benefit of such a study design, we developed a method that automatically retrieves relevant data from PubMed abstracts in order to generate prior probabilities of a genome-wide investigated variants being involved in a specific disease, and subsequently incorporates this data with the association results from GWAS using the BFDP framework [5], the Adjusting Association Priors with Text (AdAPT) method. AdAPT was subsequently applied in a GWAS of oral cancer (OC) [6]–[10].

## Results

### Power calculations for BFDP and p-values

**Figure 1:**
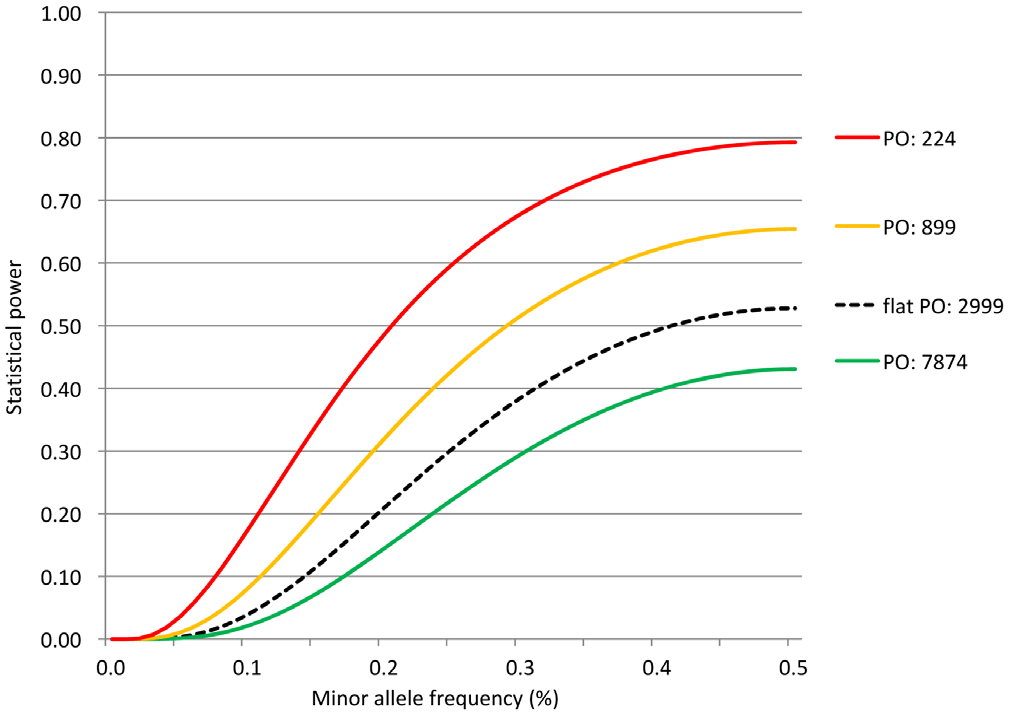
Comparison of the statistical power for three categories of prior odds for the null hypothesis when evaluating the noteworthiness of SNPs by BFDP. These power calculations assume an evaluation of 300,000 SNPs of which 100 are truly associated with the outcome and distributed evenly across three prior categories, respectively. The overall distribution of SNPs across the three prior categories is assumed to be [87.5%; 10%; 2.5%]. Flat PO assumes one single prior category.

As described by Wakefield [3], [11] the BFDP estimate can be used as means of evaluating and reporting noteworthy associations in its own right. However, we envisage a wider adoption of a hybrid, two-phase study design, in which SNPs that are deemed sufficiently “noteworthy” according to their BFDP estimates are chosen for replication in an independent study population and evaluated using the replication p-values. For instance, adopting a BFDP cut-off of 0.8 when selecting SNPs for replication implies that a false non-discovery is four times as costly as a false discovery, or that we expect on average one out of five SNPs chosen for replication to be associated with the disease. False non-discovery includes any “true” susceptibility SNP present in the dataset that did not attain a BFPD of below 0.8. Here we consider true susceptibility SNPs being associated with the phenotype of interest in statistically robust and reproducible manner, although do imply functional causality. In order to evaluate the statistical power of selecting susceptibility SNPs of oral cancer using our case-control series of 791 cases and 7,012 controls, we evaluated the statistical power according to (equation [eq.] 9, see Statistical analyses). These power calculations were based on 300,000 SNPs being evaluated in the GWAS, that 100 true susceptibility SNPs of oral cancer were included in the data set and evenly distributed across the prior categories (i.e. N* = 100, N_1_* = N_2_* = N_3_* = 33.3). We considered three prior categories (*J = 3*) and the overall SNPs in the GWAS being distributed as *C_1_ = 0.875, C_2_ = 0.10, and C_3_ = 0.025*. We can calculate the prior odds of the null hypothesis for the three prior categories under these assumptions according to (eq. 7) which gives *PO_1_ = 7874, PO_2_ = 899, and PO_3_ = 224*. The statistical power for achieving a BFDP of 0.8 for SNPs with an OR of 1.25 in each of the three prior categories is shown in Figure 1. For comparison, we include the power for using BFDP assuming the same N* but with all SNPs assigned the same prior. Under these assumptions the power to detect associated SNPs in *C_3_* or *C_2_* is increased, while sacrificing some power for those in *C_3_*. This demonstrates the potential benefits of adopting such a Bayesian framework in GWAS, if the categories and their priors are appropriately chosen.

We also included supplementary power calculations in Figure S1 by varying the assumed number of true susceptibility SNPs.

### Validation of AdAPT methodology

In order to perform an initial *proof-of-principle* evaluation of the AdAPT–BFDP method, we applied it to data from our previously reported lung cancer GWAS [12]. Firstly, we used the AdAPT web service to generate prior probabilities for SNPs based on the medical literature (see Material and methods). The keywords were grouped by priority, the first group including overarching words that are specific to lung cancer, e.g. ‘lung cancer’ and ‘lung carcinoma’, the second group included more general words specifically relevant to lung cancer, e.g. ‘smoking’, ‘nicotine’, ‘non-small cell carcinoma’, and the third group included more generic words that are not exclusively important for lung cancer, but for cancer in general, e.g. ‘carcinogen’, ‘DNA damage’, ‘neoplastic’, ‘apoptosis’. We subsequently searched through all Pubmed abstracts for each gene and assigned prior odds according to (eq. 7). We only included literature published before the date of the first lung cancer GWAS [12] in order to avoid bias.

Secondly, we split our original lung cancer GWAS into a series of smaller subsets to simulate GWAS with lower statistical power. This was performed by randomly selecting (equal distributions of cases and controls) 50% and 75% of the total data series 100 times. GWAS analysis for all subseries was then carried out and results ranked by p-value and by BFDP using priors estimated using the AdAPT web service. We compared the ranking by BFDPs and p-values within each subseries (50%, 75% or 100%) for five susceptibility variants identified by lung cancer GWAS that have been independently replicated in multiple studies (Table 1) [12]–[14]. Out of these five SNPs, four were assigned elevated priors that resulted in clear improvements in ranking when using the BFDP estimates compared to the p-values. For instance, when sampling 75% of the complete data set the rs401681 SNP on 5p15.33 was ranked at 2709 by p-value and at 664 by BFDP.

**Table 1:** Comparison of GWAS ranking of validated lung cancer susceptibility SNPs by p-values and BFDPs for known susceptibility loci of lung cancer.

SNP ID/			Ranking^4^			
Gene region/	OR (95% CI)^1^/		By p-values^5^		By BFDPs^7^	
Locus	Pr(*H_1_*)^2^	Sampling^3^	Median (Range)^6^	Mean (Stdev)	Median (Δ, Range)^6^	Mean (Δ, Stdev)
**rs1051730**	1.30 (1.19–1.43)	50%	941 (8–32308)	8604 (25454)	192 (Δ: −749, 3–7283)	1779 (Δ: −6825, 4772)
*CHRNA3*	1.4×10^−3^ (top)	75%	10 (2–1240)	312 (1722.6)	3 (Δ: −7, 1–293)	77 (Δ: −235, 421)
15q25.1		100%	2	2	2 (Δ: 0)	2 (Δ: 0)
**rs4324798**	1.44 (1.22–1.69)	50%	7305 (167–121021)	26429 (46409)	16542 (Δ: 9237, 420–133949)	35963 (Δ: +9534, 44376)
N/A	2.0×10^−4^ (low)	75%	824 (11–8320)	3427 (14488)	1450 (Δ: 626, 21–14871)	4917 (Δ: +1490, 15611)
6p22.1		100%	4	4	5 (Δ: 1)	5 (Δ: 1)
**rs401681**	0.84 (0.77–0.92)	50%	24870 (427–173666)	47681 (57625)	5416 (Δ: −19455, 82–35587)	9997 (Δ: −37684, 11336)
*TERT/CLPTM1L*	1.4×10^−3^ (top)	75%	2709 (38–25157)	8963 (22286)	664 (Δ: −2045, 13–6017)	2111 (Δ: −6852, 4706)
5p15.33		100%	73	73	30 (Δ: −43)	30 (Δ: −43)
**rs2736100**	1.18 (1.08–1.29)	50%	17573 (231–168022)	40226 (53442)	3864 (Δ: −13709, 46–35187)	8809 (Δ: −31417, 10852)
*TERT/CLPTM1L*	1.4×10^−3^ (top)	75%	2317 (142–38366)	9966 (21009)	581 (Δ: −1736, 27–9771)	2482 (Δ: −7484, 4942)
5p15.33		100%	76	76	32 (Δ: −44)	32 (Δ: −44)
**rs3117582**	1.31 (1.13–1.52)	50%	19589 (185–224392)	50985 (68903)	2850 (Δ: −16739, 45–20856)	5825 (Δ: −45160, 6601)
*BAT3*	1.4×10^−3^ (top)	75%	2648 (57–44765)	11419 (23087)	482 (Δ: −2166, 15–6513)	1647 (Δ: −9772, 2792)
6p22.33		100%	121	121	34 (Δ: −87)	34 (Δ: −87)

1) Odds ratios were estimated based on the complete dataset (100%).

2) Pr(H_1_) refers to the prior probability of the alternative hypothesis (prior probability of SNP being associated with lung cancer) and was calculated based on the AdAPT webervice being run on Entrez gene riftexts and Pubmed Abstracts, respectively. The priors were calculated in three categories (low/mid/high). See further details on the statistical framework for performing these calculations in the Methods section.

3) 50% and 75% of data were randomly sampled from the complete dataset 100 times.

4) For each randomly sampled sub-dataset we performed logistic regression and estimated odds ratios along with 95% confidence intervals, p-values and approximate bayes factors. These were subsequently used to estimate BFDPs in order to compare the ranking of known susceptibility SNPs of lung cancer using two ranking methods, by p-values and by BFPDs.

5) P-values were estimated using logistic regression models.

6) Median and mean ranking were based on the results from 100 randomly sampled datasets, Δ indicates the change in ranking compared to p-value based ranking, the range refers to the highest and lowest ranking observed, respectively.

7) BFDP (PubMed abstracts) were calculated using priors that were estimated by running the AdAPT web service on Pubmed abstracts (published before January 2008).

### Novel genome-wide association analyses

We subsequently performed a GWAS of oral cancer. This scan followed a two phase design, with the association results of the genome-wide discovery phase ranked by AdAPT-BFDPs.

#### Discovery phase

In the discovery phase, after quality control, genome-wide analysis was carried out in 791 cases and 7,012 controls. Q-Q plot analysis did not indicate any notable inflation overall (λ_inflation_ = 1.04), suggesting that hidden population substructures had little or no impact on the results of the genome-wide analysis (Figure S2). AdAPT was employed based on Pubmed abstracts, using key words relevant to oral cancer (Table S1) in a manner comparable to the lung cancer experiment outlined above. Out of 293,211 evaluated SNPs, 149,998 were grouped as *C_1_*, 137,576 were grouped as *C_2_*, and 6,637 were grouped as *C_3_*. We evaluated the individual SNP BFDP estimates using a basic sensitivity analysis approach by three distinct set of assumptions regarding the number of true susceptibility SNPs, namely N* = 50, N* = 100, and N* = 500. We applied a BFDP threshold of 0.80 for selecting SNPs for replication, and six SNPs met this criterion for all N* (Table 2). Because we had already evaluated and confirmed the 6^th^ ranked SNP (rs1789924, *AHD1C*) in a previous study of overall UADT cancer (Table 2) [11], five SNPs were selected for replication. These SNPs included rs1888732 on 1p22.3 (log additive odds ratio [OR_trend_] = 0.70, 95% confidence interval [95% CI]:0.61–0.81, *BFDP_100_* = 0.06), rs3130559 on 6p21.33 (OR_trend_ = 0.76, 95% CI: 0.65–0.88, *BFDP_100_* = 0.57), rs10801805 on 1p22.2 (OR_trend_ = 1.30, 95% CI: 1.16–1.46, *BFDP_100_* = 0.58), rs991316 on 4q23 (OR_trend_ = 0.81, 95% CI: 0.72–0.91, *BFDP_100_* = 0.62), and rs10008621 on 4q35.2 (OR_trend_ = 0.72, 95% CI: 0.60–0.86, *BFDP_100_* = 0.66).

**Table 2:** Summary results for the six SNPs selected for replication in oral cancer GWAS. Ranking was based on the Bayesian False Discovery Probability (BFDP).

		Discovery phase						Replication phase		
Locus/	SNP ID/	Cases/controls: 791/7012^a^						Cases/controls: 1046/2131^b^		
Gene region	Alleles: Freq.^c^	Odds ratio (95% CI)^d^	*P*-value^d^	Prior^e^	*P* rank^f^	BFDP (range)^g^	BFDP rank^h^	Odds ratio (95% CI)^i^	*P*-value^i^	*P*-het^j^
**1p22.3**	**rs1888732**	0.70	2.3×10^−7^	2.2×10^−4^	1	0.06	1	0.92	0.21	3.5×10^−3^
*LMO4*	A/G: 0.74/0.26	(0.61–0.80)				(0.01–0.11)		(0.81–1.05)		
**6p21.33**	**rs3130559**	0.76	2.0×10^−4^	5.0×10^−3^	68	0.57	2	1.1	0.21	7.0×10^−4^
*PSORS1C1*	C/T: 0.79/0.21	(0.65–0.88)				(0.20–0.72)		(0.95–1.28)		
**1p22.2**	**rs10801805**	1.30	6.4×10^−6^	2.2×10^−4^	2	0.58	3	0.99	0.89	1.1×10^−3^
*N/A*	G/A: 0.65/0.35	(1.16–1.46)				(0.21–0.73)		(0.88–1.12)		
**4q23**	**rs991316**	0.81	2.2×10^−4^	5.0×10^−3^	76	0.62	4	0.84	2.5×10^−3^	0.6
*ADH1C/ADH7*	C/T: 0.54/0.46	(0.72–0.91)				(0.24–0.77)		(0.75–0.94)		
**4q35.2**	**rs10008621**	0.72	3.3×10^−4^	5.0×10^−3^	112	0.66	5	0.98	0.79	0.02
*FAT1*	C/T: 0.87/0.13	(0.60–0.86)				(0.27–0.79)		(0.81–1.18)		
**4q23**	**rs17899247**	1.23	3.0×10^−3^	5.0×10^−3^	98	0.67	6	Replicated previously		
*ADH1C*	C/T: 0.59/0.41	(1.10–1.38)				(0.29–0.80)		by McKay et al.^11^		

a) Total number of cases and controls included in the final GWA analysis (Table S2).

b) Total number of cases and controls included in the replication analysis.

c) Major and minor alleles, with corresponding allele frequencies in controls.

d) OR, 95% CI and p-values were estimated for the per-rare-allele log-additive genetic model by unconditional logistic regression, adjusting for sex and country (see methods).

e) Prior probability of association (prior for the alternative hypothesis H_0_) based on the ADAPT literature search (see methods).

f) GWAS ranking based on p-values.

g) The Bayesian False Discovery Probability (BFDP) was estimated based on the association results and the prior probability of association (see methods). The point BFDP estimate corresponds to 100 true susceptibility SNPs assumed to be included in the dataset that are evenly distributed across the prior categories. The range refers to a sensitivity analysis of the BFDP by varying the assumed number of true susceptibility SNPs in the dataset. The bottom and upper boundaries were estimated by assuming 500 and 50 true susceptibility SNPs, respectively.

h) GWAS ranking based on BFDP estimates.

i) OR, 95% CI and p-values were estimated for the per-rare-allele log-additive genetic model by unconditional logistic regression, adjusting for sex and study center (see methods).

j) P-heterogeneity indicates differences in OR between the discovery and replication phases, and was derived from the Cochran's Q test.

#### Replication analysis

**Figure 2:**
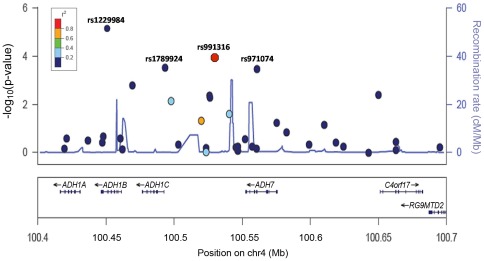
Association results of the SNPs included in the GWAS of oral cancer (by p-values), pair-wise r^2^ estimates with rs991316, and recombination rates, for SNPs in the *ADH* gene region on 4q23. P-values indicating the strength of association for each SNP in the GWAS with oral cancer are shown on the −log10 scale (left Y-axis), against their positions on chromosome 4 (Build 36.3). The color of each point and SNP represent the degree of linkage disequilibrium (r^2^) with rs991316 according to HapMap phase II CEU data. Highlighted in the figure are rs1229984, rs1789924 and rs971074, which have been reported to be associated with UADT cancers previously, as well as the rs991316 SNP which was discovered to be associated specifically with oral cancer in the current study. rs1229984 was not genotyped, nor tagged by a proxy variant on the HumanHap300 BeadChip but was genotyped by Taqman assay in the same samples from Central Europe and ARCAGE studies as included in the discovery phase of current GWAS, and r^2^ between rs1229984 and rs991316 was estimated in the 3,513 controls from Central European and ARCAGE studies. Recombination rates across the region are shown by the light blue line plotted against the right y axis. Genes in the region are represented with arrow heads indicating the direction of transcription.

**Figure 3:**
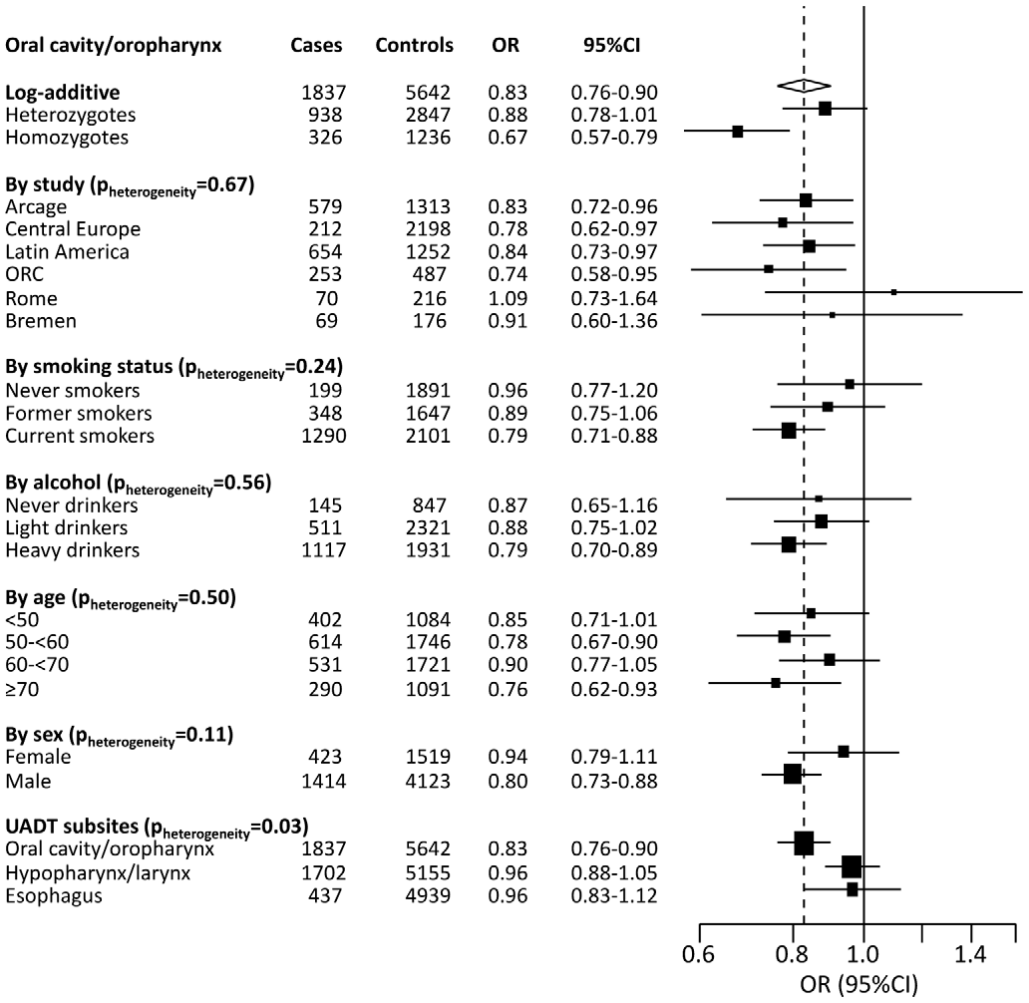
Forest plot showing overall and stratified association results of the rs991316 SNP with oral cancer (oral cavity and oropharyngeal cancer). a) Apart from the OR for CT heterozygotes and TT homozygotes, which were estimated relative the major CC homozygotes, all OR and 95% CIs were estimated using the log-additive model, adjusting for age, gender and center. All subjects from the genome-wide and replication phases with available co-variates were included in this analysis (not generic controls). The overall OR for cancers of oral cavity and oropharynx is shown by the dotted vertical line. b) P for heterogeneity indicates differences in OR between strata and was derived from the Cochran's Q test. c) Never drinkers were subjects that either reported 0 g alcohol intake per day, or reported being never drinker, light drinkers consumed >0 and <6.06 g alc./day, intermediate drinkers consumed >6.06 and <46.3 g alc./day, and heavy drinkers consumed >46.3 g alc./day. d) Hypopharynx, larynx, and esophagus cases were not included in the analyses above.

After quality control and statistical analysis within the replication series, only rs991316 displayed a statistically significant association with oral cancer risk (*per-rare-allele log additive p-value [p_trend_]* = 2.5×10^−3^, Table 2). A graph of −log_10_ p-values and pair-wise r^2^ estimates for SNPs included in the GWAS phase of the *ADH* gene region is given in Figure 2. The OR compared to the major homozygotes of the combined dataset (GWAS+replication data) were 0.88 (95% CI: 0.78–1.01) for the heterozygotes, and 0.67 (95% CI: 0.57–0.79) for the minor homozygotes. The OR associated with having one additional rare allele (log-additive model) was 0.83 (95% CI: 0.76–0.90), and this association was independently replicated (*p_trend_*<0.05) in the two largest replication studies (The Latin America and ORC studies, Table S1, *p_heterogeneity_* = 0.67). The rs991316 SNP is located in a region of 4q23 which includes several genes encoding different *alcohol dehydrogenase* (*ADH*) subunit genes, i.e. the *ADH6*, *ADH1A*, *ADH1B*, *ADH1C*, and *ADH7* genes. In the AdAPT literature search, two genes were assigned as potentially relevant for the rs991316 SNP, *ADH1C* and *ADH7*, located approximately 49 kb centromeric and 11 kb telomeric of rs991316, respectively. SNPs in this region (i.e. rs1229984 [*ADH1B*], rs1789924 [*ADH1C*] and rs971074 [*ADH7*]) have previously been associated with overall UADT cancer. However, rs991316 was poorly correlated with rs1229984, rs1789924 and rs971074 (r^2^<0.05), and conditioning the risk analysis on these SNPs did not notably affect the OR of rs991316 (OR_adjusted_<0.84). Furthermore, we investigated if rs991316 was also associated with other UADT subsites apart from oral cancers, but stratified analysis revealed that the risk effect of rs991316 were confined to oral cancer (oral cavity and oropharynx), but not cancers of the hypopharynx, larynx or esophagus (*p_heterogeneity_* = 0.03, Figure 3). Taken together, these results suggest that the rs991316 SNP is associated specifically with oral cancer, but not with other UADT cancers within this population, and that the association is independent of previously detected susceptibility SNPs of UADT cancer in this region. Furthermore, this heterogeneity in risk effects between oral and other UADT cancers may also explain why this variant was not detected in our original GWAS of overall UADT cancer.

### Stratified analyses

We conducted further stratified risk analyses for rs991316 (Figure 3), but apart from the heterogeneous risk effect on oral cancers overall, compared to other UADT cancers (*p_heterogeneity_* = 0.03), no clear effect modifications were observed (*p_heterogeneity_*>0.10). Some suggestive evidence for effect modification by gender was observed (*p_heterogeneity_* = 0.11), the association with risk being stronger among men. Some indications of tobacco smoking and alcohol intake also modifying the association with risk was also observed, with risk becoming more pronounced with higher levels of smoking and drinking, but formal tests did not support interaction (*p_interaction_* = 0.15 and 0.10 for smoking and alcohol intake, respectively). Further adjusting the main rs991316 risk analysis for drinking and smoking did not modify the OR estimates (data not shown).

## Discussion

We report a method for retrieving information from the text-based medical literature and estimating prior probabilities of association for all SNPs investigated in GWAS, the Adjusting Association Priors with Text (AdAPT) method. The priors can subsequently be incorporated with association results into a Bayesian measure of the noteworthiness of association for each SNP to disease association.

One of the main arguments for conducting GWAS is that the approach is agnostic, thus offering the ability to detect novel susceptibility loci without inferring prior beliefs regarding the importance of specific genes (e.g. compare with the candidate gene approach). However, many genetic susceptibility variants detected in GWAS reside near plausible candidate genes, and the AdAPT-BFDP method gives an opportunity to leverage this information in an automatic fashion. AdAPT automatically searches through PubMed abstracts for relevant prior evidence of involvement in the disease in question. Coupled with the BFDP statistical framework, AdAPT incorporates the prior information with the association results from the GWAS, thus giving SNPs near genes previously implicated in the disease of interest a higher ranking.

We initially validated AdAPT-BFDP based on GWAS data of lung cancer and noted that validated susceptibility SNPs were consistently ranked higher by AdAPT-BFDP estimates than by p-values (Table 1). The method was subsequently applied in a modestly sized GWAS of oral cancer (approximately 800 cases) with poor statistical power to detect the intermediate risk effects typically observed in GWAS (≤40% statistical power for choosing a SNP for replication at p<10^−5^ assuming an OR of 1.25). By ranking the association results according to AdAPT-BFDPs and replicating the top five SNPs, we identified a novel susceptibility variant within the known oral and UADT cancer susceptibility region of the *ADH* gene cluster on 4q23. We investigated if the association of rs991316 could be explained by linkage disequilibrium with previously identified risk variants of overall UADT cancer on this locus [8], [15], including rs1229984, rs1789924 and rs971074, but their pair-wise correlations were low and conditioning the risk analysis on these SNPs did not affect the OR estimate of rs991316. Furthermore, haplotype analysis clearly showed that the variant alleles of these SNPs were located on different haplotypes (data not shown), thus further strengthening the independency inference of these susceptibility SNPs. Importantly, the rs991316 SNP was not associated with other UADT cancers (Figure 3), hence supporting the notion of a novel association, as evidence for association with other UADT cancer sites has been noted with the previously detected susceptibility SNPs.

We acknowledge that any method that takes non-curated prior information into account in association studies is imperfect and subject to various biases [2], and the AdAPT-BFDP method has several limitations. As AdAPT searches PubMed abstracts for the presence of pre-assigned keywords and semantically related concepts, the final result (ranking by BFDPs instead of p-values) will privilege SNPs in the proximity of genes that have been studied in relation to the endpoint of interest. As shown in Figure 1, there is a loss in statistical power for SNPs for which no relevant prior information is available. This penalty imposed on novel genes and gene deserts would seem contradictory to the agnostic nature of a GWAS, even though strong association signals will remain highly ranked using either method.

The method is also sensitive to the assumed number of truly associated SNPs (N*). Increasing this number will allow more SNPs to pass the BFDP threshold, but it will not change their ranking. In a two phase design where the number of SNPs retained for the second phase is determined by the second-stage power [16], only the relative ranking in the first stage is relevant and the choice of N* is immaterial. By contrast, the proportion of truly associated SNPs in each prior category influences the ranking by changing the prior probability of association for each variant.

Further, the current implementation of AdAPT uses a relatively simple text mining algorithm and, as such, remains relatively crude. For example, it does not take the frequency of key-word matches into account when assigning the priors, nor does it take into account if individual studies report positive or negative study results. Such strategies will be evaluated in future implementations of AdAPT, as well as methods aiming to expand keywords into semantically related concepts. In addition, AdAPT currently assigns individual SNPs to genes simply based on their location, e.g. if they are within 50 kb from a given gene. Here it would seem useful to also take linkage disequilibrium into account, as has been implemented in the Gene Relationships Across Implicated Loci (GRAIL) methodology [17]. Similarly to AdAPT, GRAIL uses text mining of PubMed abstracts to prioritize SNPs in GWAS, but does so by identifying genes that are functionally related to multiple other genes to a higher extent than what would be expected by chance. While the GRAIL methodology has the advantage of not requiring the phenotype of interest to be studied in relation to a particular gene before, nor does it benefit when such information is available. Hence, it seems both the AdAPT and GRAIL methodologies may prove useful together, or on their own, in prioritizing SNPs from initial GWA scans for further follow-up.

Importantly, we envisage using the AdAPT-BFDP method as complementary tool - rather than as a replacement - to the more traditional GWAS approach (i.e. p-value ranking), e.g. by initially using p-value based ranking to detect genetic loci in an agnostic manner, and subsequently, the AdAPT-BFDP methodology to further leverage the data with a potential to detect variants that may otherwise be overlooked. While it is generally not recommended to conduct underpowered studies, the AdAPT-BFDP method may also assist detection of susceptibility loci when the statistical power is poor, for instance in stratified genome-wide analysis such as in the underpowered oral cancer GWAS. Indeed the rs991316 SNP was ranked 76^th^ by p-values, and this susceptibility SNP would not have been included in the replication phase had we adopted to replicate only the very top ranked variants by p-values. Furthermore, if the number of SNPs selected for replication had been sufficiently deep to include rs991316, the statistical evidence for replication (*P_trend_* = 2.5×10^−3^) would not have been deemed noteworthy after adjustment for multiple testing in the replication phase (i.e. a Bonferroni adjusted significance threshold of p = 0.0007). Hence, it appears that the AdAPT-BFDP strategy assisted the detection and validation of the rs991316 variant. The AdAPT process can also be easily adapted to provide prior information of overall genes rather than individual SNPs, and as such, may also be useful in genomic applications, such as exome or genome based sequencing studies. Furthermore, several sources of information could potentially be included within the Bayesian framework, for example pathway ontology databases, other text based methods including GRAIL, or complementary experiments such as genome-wide eQTL analysis [18].

### Conclusions

This study confirms that it is possible to incorporate comprehensive prior information in an automated fashion to assist in prioritizing SNPs in GWAS for further follow-up, in this case from the text-based medical literature using the AdAPT-BFDP methodology. In support of this, we report a novel susceptibility SNP of oral cancer in the *ADH* gene region of 4q23, which was associated with risk independently of previously identified risk SNPs of overall UADT cancer in this region. We have made the AdAPT methodology available to the research community through a web service (url: http://services.gate.ac.uk/lld/gwas/service/config).

## Materials and Methods

### Ethics statement

All participants gave written informed consent to participate in the study and the IARC Ethics Committee (IEC) approved this research.

### Retrieving information from the medical literature using AdAPT

In order to extract relevant information from the medical literature in a comprehensive and unbiased fashion, we developed the Adjusting Association Priors with Text (AdAPT) method. AdAPT identifies relevant PubMed abstracts for each RefSeq gene through the Entrez gene database (url: http://www.ncbi.nlm.nih.gov/gene), where all studies that have investigated a particular gene are cross referenced with PubMed. For this study, any gene within 50 000 base pairs of a SNP was mapped, together with the abstracts linked to that gene. It is also possible to use the GeneRif texts, which are short and manually annotated summaries of each research paper, in place of PubMed abstracts. These GeneRif texts are directly provided in the Entrez gene database. All relevant abstracts are subsequently mined for keywords and key concepts relating to important features of the disease or phenotype of interest, including etiological and mechanistic factors. This mining is carried out using GATE (url: http://gate.ac.uk) [19], which splits abstracts into sentences, tokenizes the sentences into individual terms, finds the part of speech for tokens, and breaks each token into its main component (morphological root). Abstracts were also mapped to UMLS concepts using MetaMap [20], [21]. Tokens and concepts were stored in a GATE Mimir index to facilitate fast retrieval and to store mapping between individual SNPs and relevant abstracts [22]. Keywords for mining were also processed with GATE to give morphological roots and the presences of these were subsequently checked in the index for each SNP.

We assigned keywords into one of three groups, G1, G2 and G3, group G1 containing words of the highest importance for the phenotype, and group G3 containing relevant, but subjectively less important words. Based on the presence of relevant keywords, each gene and proximal SNPs can logically be assigned to one of 8 possible binary combinations of G1, G2 and G3. For our purposes we defined three categories (*C_i_, i = 1,2,3*):

1. *C_1_* = {Not *G_1_*, Not *G_2_*, Not *G_3_*}
2. *C_2_* = {At least one of *G_1_, G_2_, G_3_* but not all }
3. *C_3_* = {*G_1_, G_2_, G_3_*}.

We developed a web service that allows a user to conduct key word queries over an arbitrary set of SNPs in a timely manner, e.g. a list of SNPs included on a particular genome-wide BeadChip (url: http://services.gate.ac.uk/lld/gwas/service/config). This returns a classification over all 8 possible categories, allowing further collapsing by the user. We also provide an R-script that estimates the prior probabilities for each SNP and re-ranks the GWAS results according to the BFDP estimates. This will allow investigators to freely apply the AdAPT methodology without uploading their association results online (url: http://services.gate.ac.uk/lld/gwas/service/rscript). The R-script also allows the user to redefine the grouping of prior categories.

### Statistical analysis

#### Assigning prior probabilities of association and the Bayesian false discovery probability (BFDP)

In order to estimate the Bayesian false discovery probability (BFDP), as proposed by Wakefield [5], we first need to estimate the Bayes factor, the relative likelihood of the data y under either the null hypothesis (no association, H_0_) or the alternative (SNP associated with the disease, H_1_):
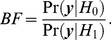
(1)Estimating the Bayes factor is computationally difficult because it requires the complete specification of both the data-generating mechanism (the likelihood), the prior distribution for *all* parameters of the model, and the calculation of multidimensional integrals. To overcome these difficulties Wakefield introduced the approximate Bayes factor (ABF). For a generic SNP, in the case of a single summary parameter ***θ*** (e.g. a log odds ratio) with estimate 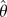, standard error 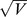, and a normal prior N(0,W) on ***θ***:
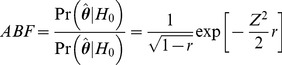
(2)Where *Z* is the normal statistic 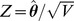 and ***r*** is the ratio between the prior and total variance (***r*** = *W*/(*V+W*)). The estimate 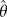 and standard error 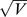 are readily available from standard regression output [5]. The Bayesian False Discovery Probability is defined as
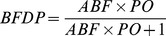
(3)where *PO* is the prior odds for H_0_. It provides a means of evaluating the noteworthiness of SNP-endpoint associations that takes both the association result and prior evidence into account [5].

In order to incorporate the prior information gained in the AdAPT literature search (*C*) into the *BFDP* we need to estimate the prior odds of the null hypothesis given the AdAPT results as
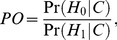
(4)where C is a generic prior category. Each SNP evaluated in a GWAS will fall into one of *J* prior categories (*C_j_*), i.e. as shown above. In order to estimate (eq. 4) we need to compute Pr(*H*_0_|*C_j_*) and Pr(*H*_1_|*C_j_*), *j* = 1,…,*J*. We can write the prior probability for a SNP to fall into prior category *j* as
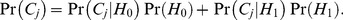
(5)We assume that *N* SNPs are being evaluated in the GWAS, and that the number of SNPs which are truly associated with the endpoint in each prior category is 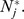 We can then write the overall probability of the alternative hypothesis as 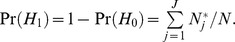 The AdAPT literature search will provide the overall distribution of SNPs across the prior categories, *N_j_*, and we can calculate, as well as 
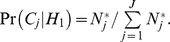
 From (eq. 5) we have
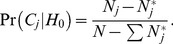
(6)According to Bayes theorem (with *C_j_* acting as the data) we can write the prior probabilities of the null and alternative hypotheses given the AdAPT search as 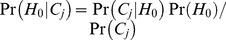 and 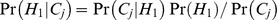 respectively, which gives the prior odds of H_0_ given a SNP in prior category *j*.
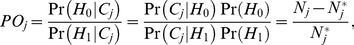
(7)the difference between the total number of SNPs in category *j* and the number of truly associated SNPs in category *j*, divided by the number of truly associated SNPs in category *j*. We can now calculate the *BFDP* which not only takes the strength of association into account, but also the statistical power and the prior probability of association according to the AdAPT literature search. In making a final decision we may choose to reject *H_0_* if the BFDP falls below a threshold **γ**.

In order to evaluate the statistical power *q* to achieve a *BFDP* of **γ**, given ***y*** and *PO_j_* we need to evaluate 
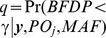
 which is equivalent to
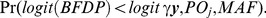
(8)If we substitute *BFDP* for the expression in (eq. 3) we have
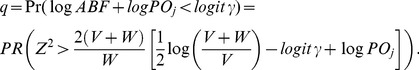
(9)Here *Z* ^2^ is a non-central χ^2^ with 1 degree of freedom and non-centrality parameter *θ* ^2^/*V*, which is equivalent to 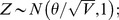 under *H*_0_ we have the special case 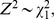 i.e. a chi-squared random variable with 1 degree of freedom.

### Frequentist analysis

The association between each genetic variant and cancer risk was estimated using the per-rare-allele log-additive genetic model. Odds ratios (OR) were estimated by unconditional logistic regression, adjusting for gender and country of recruitment (GWAS), or for gender, age and study center (replication).

For the GWAS, quantile-quantile plot analysis of p-values was conducted to evaluate if the genome-wide analysis was subject to systematic bias. These analyses were conducted using PLINK and R [23].

In the replication phase, data were stratified by study group and the homogeneity of OR between strata was tested using Cochran's Q-test. These analyses were conducted using SAS 9.2 software, and all p-values were two-sided.

### Study population

An initial proof-of-principle analysis was carried out using data from a GWAS of lung cancer including 1,989 cases and 2,625 controls. This study was conducted in six central European countries and of which details have been previously published [12].

Subsequently, we conducted a novel analysis using data generated in an oral cancer GWAS. Genome-wide genotyping was performed in two European based multi-centre case-control studies (Table S2), the International Agency for Research on Cancer (IARC) central Europe (CE) study conducted from 2000 to 2002, in 6 centers from 5 countries [8], [12], [24] and the ARCAGE (**A**lcohol-**R**elated **C**ancers **a**nd **G**enetic susceptibility in **E**urope) multi-centre case control study conducted by IARC from 2002 to 2005 in 12 centers from 9 European countries [8], [12], [25].DNA of sufficient quality and quantity for genome-wide genotyping was available for a total of 859 oral cancer cases (squamous cell carcinomas) and 3,999 controls from these two studies. Oral cancer cases included cancer of the oral cavity and oropharynx. We additionally included 3,641 generic controls to further increase the statistical power. These generic controls included: 1,385 individuals from the 1958 birth cohort (Wellcome Trust case control consortium) [26], as well as 1,823 French and 433 Norwegian controls genotyped by the Centre National Genotypage (CNG Evry France). The replication series consisted of 1,046 oral cancer cases (squamous cell carcinomas) and 2,131 controls from 4 case-control studies (Table S2) [27], [28].

### Genotyping

#### Discovery phase (genome-wide genotyping)

The CE study and the ARCAGE study, as well as the generic controls from Norway and France, were genotyped using the Illumina Sentrix HumanHap300 BeadChip at the Centre d'Etude du Polymorphisme Humain (CEPH) and the CNG as previously described [12], [29]. The generic UK controls were genotyped at the Wellcome trust Sanger Institute using the Illumina HumanHap 550 BeadChip [26].

We conducted systematic quality control steps on the raw genotyping data. SNPs with a genotype call rate of less than 95%, as well as individuals where the overall genotype completion rate was less than 95%, were excluded. We further excluded SNPs for which the genotype distribution clearly deviated from that expected by Hardy-Weinberg Equilibrium (HWE) among controls (p-value<10^−7^). Also excluded were individuals for which we observed discrepancies between reported gender and gender based on genotypes, as well as individuals with unlikely heterozygosity rates across genetic variants on the X chromosome. Those genotyped were restricted to individuals of self-reported European ethnicity. To further increase the ethnic homogeneity of the series, we used the program STRUCTURE to identify individuals of mixed ethnicity [30]. Using a subseries of 12,898 genetic variants from the HumanHap300 BeadChip panel evenly distributed across the genome and in low linkage disequilibrium (LD) (r^2^<0.004) [31], we estimated the genetic profile of the study participants compared with individuals of known ethnic origins (the Caucasian, African and east-Asian individuals genotyped by the HapMap project). We excluded 34 individuals because of some evidence of ethnic admixture, indicating that the extent of admixture within the central Europe and ARCAGE study centers is limited.

#### Replication phase (TaqMan genotyping)

Replication genotyping was performed on the 1,046 oral cancer cases and the 2,131 controls using the TaqMan genotyping platform at IARC. The robustness of the TaqMan assays (primers and probes are available upon request) were confirmed by re-genotyping the CEPH HapMap (CEU) trios and confirming concordance with HapMap genotypes. All TaqMan assays were found to perform robustly and genotype concordance rates for duplicate samples were above 99.5%.

## Supporting Information

Figure S1 **Comparison of the statistical power when evaluating the noteworthiness of SNPs by BFDP.** These power calculations assume an evaluation of 300,000 SNPs of which 20 (Figure S1A) and 500 (Figure S1B) are truly associated with the outcome and distributed evenly across three prior categories, respectively. The overall distribution of SNPs across the three prior categories is assumed to be [87.5%; 10%; 2.5%]. Flat PO assumes one single prior category.
(TIF)

Figure S2 **Quantile-quantile plot for** ***p*** **-values on −log10 scale.**
(TIF)

Table S1 **Keywords used to generate AdAPT priors in the oral cancer GWAS.**
(DOC)

Table S2 **Participating studies.**
(DOC)
