# Supplementary figures and images for: Using Prior Information from the Medical Literature in GWAS of Oral Cancer Identifies Novel Susceptibility Variant on Chromosome 4 - the AdAPT Method
Source: PLoS One. 2012 May 25;7(5):e36888. doi: 10.1371/journal.pone.0036888 (PMC3360735; doi:10.1371/journal.pone.0036888)

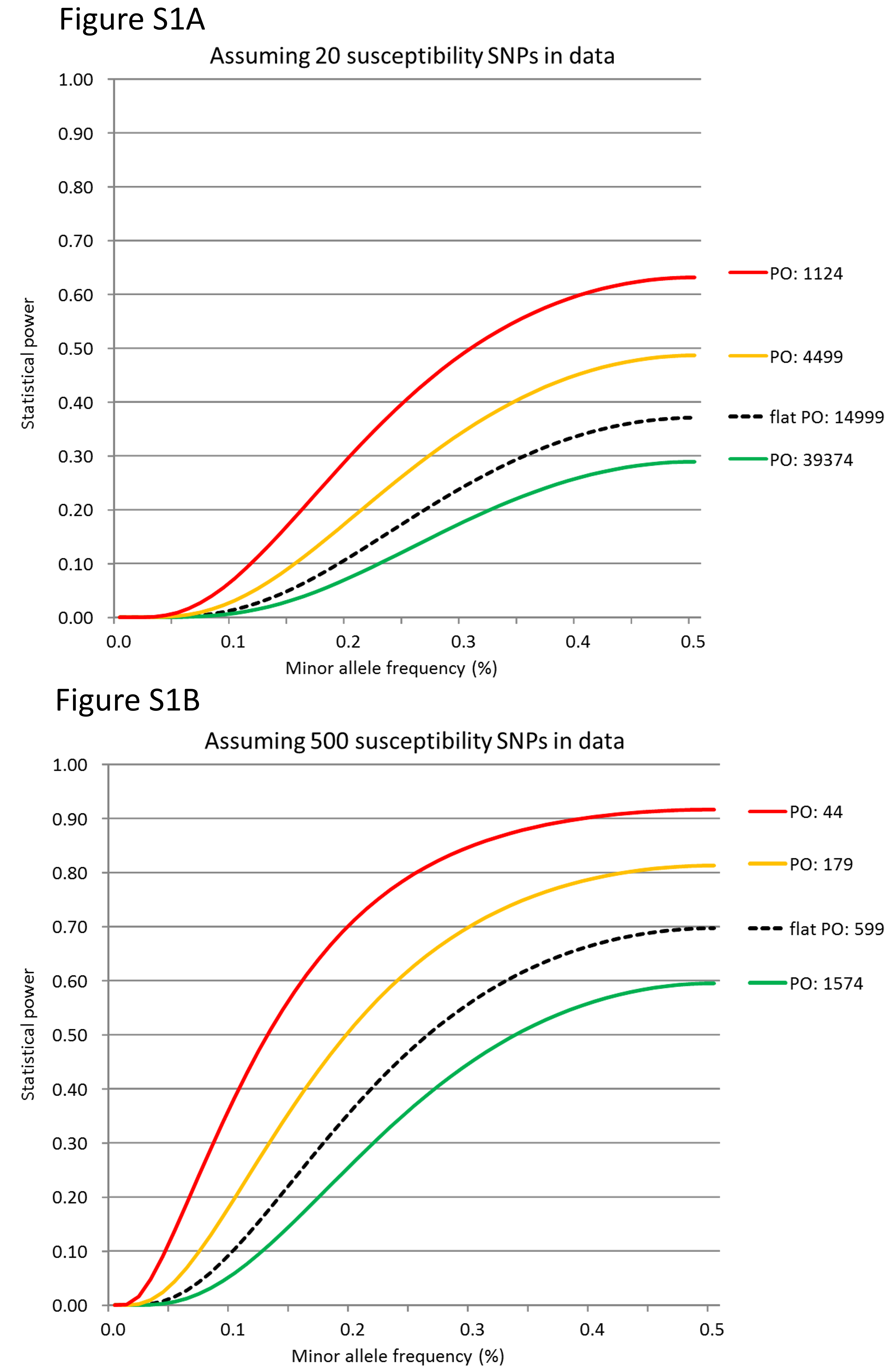

Supplement: Figure S1 — Comparison of the statistical power when evaluating the noteworthiness of SNPs by BFDP. These power calculations assume an evaluation of 300,000 SNPs of which 20 (Figure S1A) and 500 (Figure S1B) are truly associated with the outcome and distributed evenly across three prior categories, respectively. The overall distribution of SNPs across the three prior categories is assumed to be [87.5%; 10%; 2.5%]. Flat PO assumes one single prior category. (TIF) [file pone.0036888.s001.tif]

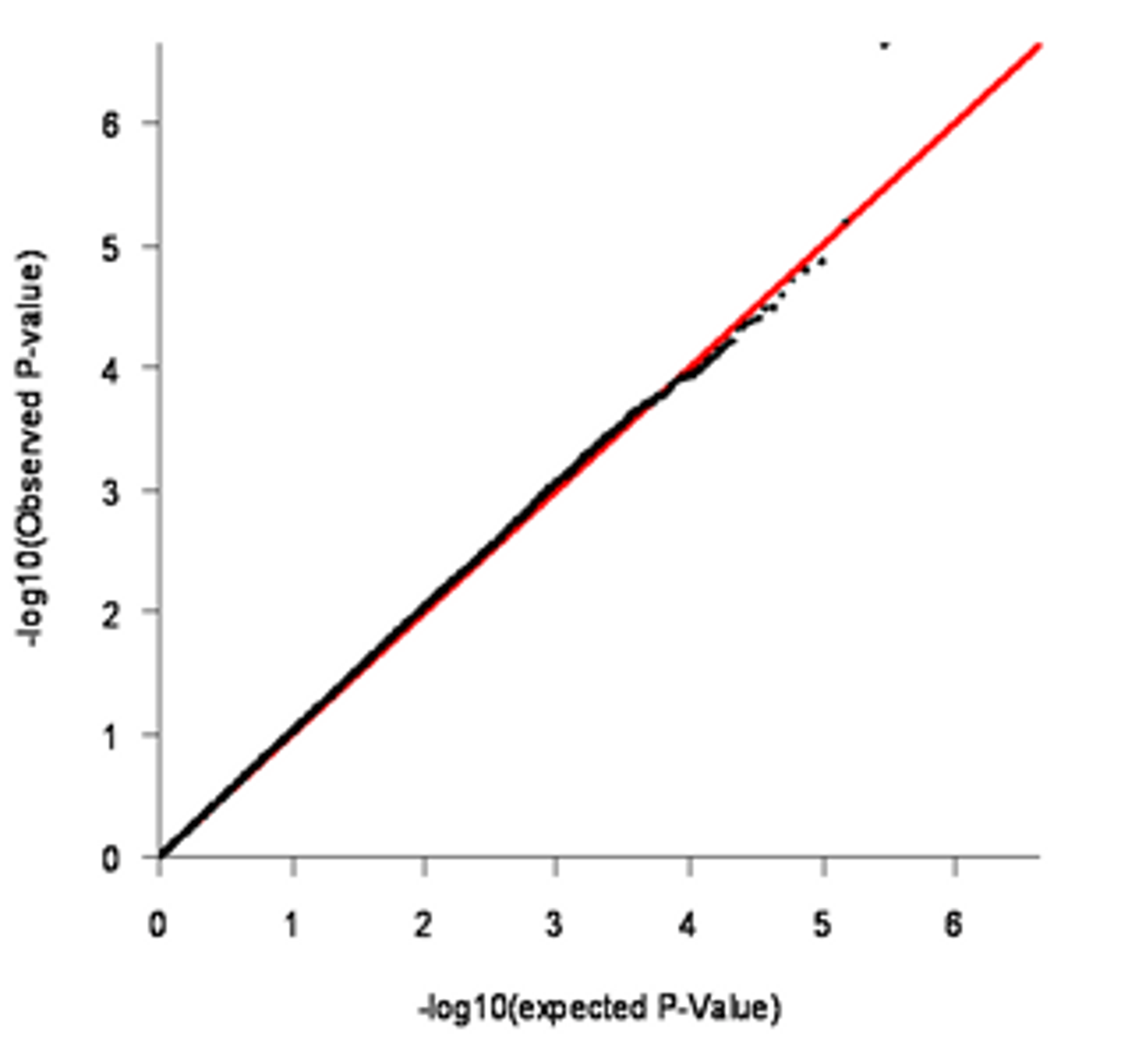

Supplement: Figure S2 — Quantile-quantile plot for p -values on −log10 scale. (TIF) [file pone.0036888.s002.tif]
