# Supplementary material for: Using Prior Information from the Medical Literature in GWAS of Oral Cancer Identifies Novel Susceptibility Variant on Chromosome 4 - the AdAPT Method
Source: PLoS One. 2012 May 25;7(5):e36888. doi: 10.1371/journal.pone.0036888 (PMC3360735; doi:10.1371/journal.pone.0036888)
Supplement: Table S1 — Keywords used to generate AdAPT priors in the oral cancer GWAS. (DOC) [file pone.0036888.s003.doc]

# Supporting information

| **Table S1 | Keywords used to generate AdAPT priors in the oral cancer GWAS** | | | | | |
| --- | --- | --- | --- | --- | --- |
| **Keyword group 1** |  | **Keyword group 2** |  | **Keyword group 3** | |
| Oral cancer |  | smokeless tobacco |  | chewing tobacco | Head & neck |
| head and neck cancer |  | Nicotine |  | tongue | oral cavity |
| UADT cancer |  | Nicotine addiction |  | hypopharynx | tonsil |
| pharyngeal cancer |  | carcinogenesis |  | genome-wide association study | oropharynx |
|  |  | squamous cell carcinomas |  | human papillomavirus | genome wide association study |
|  |  | alcohol |  | oxidative stress | soft palate |
|  |  | tobacco |  | dna repair | carcinogen |
|  |  | smoker |  | upper aerodigestive tract | mouth |
|  |  | neoplasm |  | palate | cheek |
|  |  | smoking |  | genetic disease | hard palate |
|  |  | tumor |  | lip | buccal mucosa |
|  |  |  |  | gingivae | mucosal |
|  |  |  |  | gum | HPV |
|  |  |  |  | cigar | piriform fossa |
|  |  |  |  | UADT | pharynx |
|  |  |  |  | floor of mouth | cancer |
|  |  |  |  | carcinoma | genetic trait |
