# Supplementary material for: Using Prior Information from the Medical Literature in GWAS of Oral Cancer Identifies Novel Susceptibility Variant on Chromosome 4 - the AdAPT Method
Source: PLoS One. 2012 May 25;7(5):e36888. doi: 10.1371/journal.pone.0036888 (PMC3360735; doi:10.1371/journal.pone.0036888)
Supplement: Table S2 — Participating studies. (DOC) [file pone.0036888.s004.doc]

# Supporting information

| **Table S2 | Participating studies** | | | | | | | | | | | | | | |
| --- | --- | --- | --- | --- | --- | --- | --- | --- | --- | --- | --- | --- | --- | --- |
|  |  |  |  |  | **Samples with genome-wide data^a^** | | |  | **Samples used in final analysis^b^** | | |  |  |  |
| **Study name** | **Coordinating centre** | **Study design** | **Study base** |  | **Cases** | **Controls** | **Generic controls** |  | **Cases** | **Controls** | **Generic controls** |  | **Genotyping array^c^** | **Genotyping center**^c^ |
| **Stage I. Studies included in the genome-wide discovery phase** | | | | | | | | |  | | | | | |
| Central Europe (CE)^d^ | IARC | Hospital based case-control | Europe - Multicentre |  | 234 | 2,610 | - |  | 212 | 2,200 | - |  | 317K | CNG |
| ARCAGE^e^ | IARC | Hospital based case-control^f^ | Europe - Multicentre |  | 625 | 1,389 | 3,641^g^ |  | 579 | 1,313 | 3,499 |  | 317K/550k | CNG/ Sanger |
|  |  |  |  |  |  |  | **Total:** |  | **791** | **3,513** | **3,499** |  |  |  |
| **Stage II. Studies included in the replication phase** | | | | | | | | |  | | | | | |
| Latin America (LA) | IARC | Hospital based case-control | Latin America - Multicentre |  | - | - | - |  | 654 | 1,252 | - |  | TaqMan | IARC |
| IARC-oral cancer (ORC) | IARC | Hospital based case-control | Europe-Multicentre |  | - | - | - |  | 253 | 487 | - |  | TaqMan | IARC |
| ARCAGE - Bremen | Bremen Uni. | Hospital based case-control | Bremen -Germany |  | - | - | - |  | 69 | 176 | - |  | TaqMan | IARC |
| Rome | Rome Uni. | Hospital based case-control | Rome - Italy |  | - | - | - |  | 70 | 216 | - |  | TaqMan | IARC |
|  |  |  |  |  |  |  | **Total:** |  | **1,046** | **2,131** |  |  |  |  |
| *a) Only subjects that were of self-reported European ancestry were included. b) For stage I, samples with GW data that were included in the final genome-wide analysis after quality control steps (see methods). c) Genome-wide bead chip used: 317k: Illumina HumanHap300; 550k: Illumina HumanHap550. c) CNG: Centre National de Génotypage, France; Sanger Institute: the Wellcome Trust Sanger Institute, UK. d) Includes countries: Romania, Poland, Russia, Slovakia, Czech Republic. e) Includes countries: Czech Republic, Greece, Italy, Norway, UK, Spain, Croatia, Germany, France. f) 3 UK centers collected population-based controls and the other 13 centers used hospital-based controls. g) From the 1958 British birth cohort in Wellcome Trust case-control consortium, Norwegian controls and French studies.* | | | | | | | | | | | | | | |
